# Service-learning in response to the coronavirus disease 2019 pandemic: Emerging lessons from the Department of Family Medicine and Public Health at the University of Botswana

**DOI:** 10.4102/phcfm.v12i1.2455

**Published:** 2020-06-03

**Authors:** Billy M. Tsima, Tiny Masupe, Vincent Setlhare

**Affiliations:** 1 Department of Family Medicine and Public Health, Faculty of Medicine, University of Botswana, Gaborone, Botswana

## Abstract

The novel coronavirus disease 2019 (COVID-19) pandemic has disrupted many lives worldwide. Training programmes in academic institutions have also been affected by the pandemic. Teaching and learning family medicine and public health medicine in the COVID-19 era require adjustments to training activities. At the University of Botswana, the pandemic presented an opportunity to steer training programmes in the Department of Family Medicine and Public Health Medicine more towards service-learning. The department collaborated with the Ministry of Health and Wellness as well as the District Health Management teams in the national response to the pandemic as essential service providers. The increased demands for service provision were balanced with educational opportunities for trainees during the COVID-19 public health emergency. Including structured ongoing reflections for trainees involved in the COVID-19 response helps to connect service and the academic curriculum.

**Keywords:**

COVID-19; coronavirus; service-learning; pandemic; Botswana.

## Introduction

The coronavirus disease 2019 (COVID-19) pandemic has disrupted life worldwide. The public health interventions aimed at curbing the spread and impact of this disease, such as social distancing and lockdown, have resulted in disruption in academic programmes. The University of Botswana (UB) offers courses ranging from humanities to medicine. The Faculty of Medicine (FoM) programmes at UB include Family Medicine, Public Health Medicine and other disciplines. These are 4-year residency programmes that require structured learning. The FoM produces graduate doctors, specialists in family medicine and public health medicine, and specialists in other disciplines.

## Impact of coronavirus disease 2019 restrictions on academic activities

In Botswana, a state of public emergency (SOE) was declared following the reporting of three cases of COVID-19 on 02 April 2020. The SOE required that all institutions of learning should be closed and that no face-to-face teaching and learning should take place. A 28-day country-wide lockdown, which was followed by a 6-month SOE as requested by the President, to control and contain COVID-19 was effected.

The lockdown and the SOE have impacted postgraduate medical training. There is uncertainty about scheduled examinations and the academic calendar as a whole. Thus, it is impossible to set timelines for activities and to predict the return to a normal academic calendar. Early reports indicate that the clinical learning environment has also become compromised. Clinical departments have curtailed their workload to urgent and emergency care, with non-urgent and elective cases being excluded from routine service at this time. The variety and volume of clinical cases for trainees to gain competency from have been compromised. National disease control programmes in the Department of Public Health at the Ministry of Health and Wellness (MOHW), such as malaria, tuberculosis and non-communicable diseases, have been downsized so as to direct all attention to the COVID-19 pandemic. District health management teams (DHMTs) are also diverting resources towards surveillance activities, including testing of suspected cases and contact tracing. It is important for postgraduate training programmes to be responsive to the public health emergency of COVID-19 and to seize the opportunity of continuing medical education afforded by the health system as it focusses on this novel disease.

The Department of Family Medicine and Public Health Medicine at UB has been affected by the new developments that threaten the feasibility of the year’s planned academic activities. Movement restrictions and limitation of group numbers mean that academic activities requiring travel and large group meetings cannot proceed as planned. As academic and postgraduate trainees are regarded essential for the national response to the pandemic, they are increasingly occupied in service provision tailored to the demands of the evolving COVID-19 pandemic, and consequently spend less time in academic programmes. There is a need to balance the increased service provision with the academic programmes and the educational opportunities of the COVID-19 emergency.

At UB, our department offers MMED in Family Medicine and MMED in Public Health Medicine. Both of these programmes are ideally placed to serve at the frontlines of a pandemic such as COVID-19 and to model service-learning.

## Service-learning in the era of coronavirus disease 2019

Service-learning is a pedagogy of engagement wherein students address a community need by engaging in volunteer service that is connected explicitly to the academic curriculum through structured ongoing reflections.^1^ The COVID-19 pandemic thus presented an opportunity for our department to steer our training programmes further towards service-learning. Our two training programmes joined the national response towards the pandemic on two strategic fronts.

The Family Medicine programme has collaborated with the Gaborone DHMT to set up a centralised ambulatory COVID-19 screening clinic at the Sir Ketumile Masire Teaching Hospital, the academic hospital of UB. Senior registrars and clinical academic staff assist in staffing the clinic. Similarly, staff and senior registrars in two other campuses of our department are involved in screening patients for COVID-19, teaching healthcare workers on COVID-19 and developing COVID-19 patient management algorithms, in collaboration with DHMTs.

Staff and registrars in Gaborone provide service at quarantine sites by screening for COVID-19, taking specimens for testing, as well as caring for quarantined people with chronic diseases and those needing emergency medical care. Our academic staff spend time with the registrars weekly to reflect on their COVID-19 experiences, avail registrars debriefing sessions when possible and continue with the academic programme to whatever extent possible.

Aspects of clinical care for an infectious disease, such as proper personal protective equipment use (donning and doffing), patient-centred care that explores a three-stage assessment (clinical, individual and contextual)^2^ and self-care, form part of the weekly reflections. The rapidly changing knowledge surrounding the COVID-19 pandemic has also demanded knowledge synthesis from the emerging scientific literature, thus enhancing the scholarly attributes of the Canadian Medical Education Directives for Specialists (CanMEDS) framework informing our curriculum.^3^

Public health registrars and staff are collaborating with the DHMTs for conducting contact tracing and communicating prevention awareness. They are attached to the MOHW where they help formulate COVID-19 strategies and policies. They lead surveillance and contact tracing at the national level, and help in the documentation of the national response and research on COVID-19. The registrars also provide training on epidemic preparedness, infection control and use of personal protection equipment. While they are gaining valuable lessons on outbreak investigation, their programme learning has been disrupted. Mitigation of loss of programme learning includes continued supervision by attachment supervisors who also ensure attendance and assess the work done by registrars. Supervisors ensure that learning on the job continues despite COVID-19 assignments. Registrars will be evaluated for their work in COVID-19 assignments. Furthermore, following the peak period of the pandemic, our programmes will utilise time allocated to elective and self-directed learning to catch up with non-COVID-19-related training that was unavoidably missed during the pandemic.

## Conclusion

The COVID-19 pandemic presents a lifetime opportunity for learning for family medicine and public health medicine trainees. The opportunity however comes with disruption of structured learning. Innovative ways of reducing this disruption are critical. These can include credit for work done during service and use of technology.
